# Prevalence, perceived effects, and motivations for adolescent e-cigarette use: A cross-sectional study in the Paris region

**DOI:** 10.18332/tpc/225318

**Published:** 2026-07-31

**Authors:** Mathilde Gendrot, Anne-Laurence Le Faou

**Affiliations:** 1 Faculty of Medicine, Université Paris CitéParisFrance; 2 Faculty of Medicine, Sorbonne UniversitéParisFrance; 3 Outpatient Addictology Center, Georges Pompidou European HospitalParisFrance

**Keywords:** electronic cigarettes, e-cigarettes, adolescents, France, smoking

## Abstract

**Introduction:**

Adolescence is a critical period for experimentation with psychoactive substances, including electronic cigarettes (e-cigarettes). This study aimed to assess the prevalence of e-cigarette use among high school students in Paris and to explore adolescents’ motivations for use and their perceptions of its effects.

**Methods:**

This cross-sectional study, based on self-reported data collected through an online questionnaire, was conducted between January and April 2021 among students from six educational institutions in the Paris region. The anonymized survey collected demographic and health data, information on tobacco and e-cigarette use behaviors, and perceptions and motivations related to e-cigarette use. Associations between the social environment and tobacco and e-cigarette experimentation were evaluated using prevalence ratios, crude odds ratios (ORs), and adjusted ORs (AORs), with 95% confidence intervals (CIs).

**Results:**

A total of 1187 students participated (median age: 17 years; 55.5% male). E-cigarette experimentation was reported by 46.8%, current use by 18.9%, and daily use by 8.6%. The mean age at e-cigarette initiation was 14.9±1.9 years. Dual experimentation with tobacco and e-cigarettes was observed in 39.2% of participants, while 5.2% reported exclusive e-cigarette use. Curiosity was the most frequently reported motivation for e-cigarette ever users (91.4%), followed by flavor variety (75.0%), and the ability to use e-cigarettes at home without tobacco odor (63.6%). Exposure to e-cigarette use among friends was strongly associated with e-cigarette experimentation (AOR=3.68; 95% CI: 2.88–4.73). Notably, 23.4% of all respondents and 12.3% of e-cigarette ever users were unaware whether e-liquids contained nicotine. Most e-cigarette users expressed concerns about lung health (85.2%) and addiction risk (75.5%).

**Conclusions:**

E-cigarette use is prevalent among adolescents in the Paris region and is driven by curiosity, sensory appeal, and social influences. Additional studies, particularly longitudinal cohort studies, are needed to provide evidence-based information on tobacco and e-cigarette use trajectories among adolescents.

## Introduction

Adolescence represents a critical period for experimentation with psychoactive substances, including tobacco. In France, in 2024, 30.6% of high school students reported having tried tobacco at least once in their lifetime, 13.5% reported smoking in the previous 30 days, and 5.6% reported daily smoking^1^. While tobacco experimentation and cigarette smoking have declined in recent years among French adolescents, the use of electronic cigarettes (e-cigarettes) has increased^1^. Indeed, in 2024, 46.0% of French high school students reported having experimented with e-cigarettes, and 25.3% reported use within the previous 30 days^1^. In a meta-analysis of 40 studies among students (secondary school, high school, or university), the worldwide prevalence of e-cigarette ever use was estimated at 22.7%, and current use at 13.0%^2^.

E-cigarettes, which rapidly emerged as a new consumer product in the early 2010s, are battery-operated devices that generate an aerosol from a liquid solution (e-liquid) typically composed of propylene glycol or vegetable glycerin, flavorings, and, optionally, nicotine, with concentrations limited to 20 mg/mL^−1^ in Europe^3,4^. A wide range of e-cigarette devices exists, including BOX devices (square or rectangular vaporizers that can hold one or more rechargeable batteries, generally high-powered and customizable), portable-on-demand (POD) devices (compact systems in which a replaceable cartridge, referred to as a pod, containing the e-liquid and heating coil is attached to a small battery unit by a snap-fit or magnetic connection), and TUBE or pen-style devices. Compared with adults, adolescents often prefer POD systems such as Puff Bar and JUUL because they are discreet, easy to use, and available in appealing flavors, while also delivering relatively high nicotine concentrations^5-8^. Furthermore, adolescents’ motivations for e-cigarette use may differ from those of adults, who primarily cite smoking cessation, whereas adolescents more often report curiosity, appealing flavors, or performing vaping tricks^9,10^.

Although e-cigarettes have been proposed as a potential harm-reduction or smoking cessation tool, their benefit-risk balance remains debated. Nicotine exposure and the potential for nicotine addiction, especially from products marketed as nicotine-free but sometimes containing trace amounts, raise concerns about the development of dependence and the possible progression to tobacco smoking^11^. In addition, the pulmonary effects of inhaling heated propylene glycol, vegetable glycerin, and a wide range of largely unregulated flavorings remain uncertain, as these substances may degrade into irritant or carcinogenic compounds^12,13^. Moreover, the 2019 outbreak of e-cigarette or vaping product use-associated lung injury (EVALI) in the United States was linked to additives such as vitamin E acetate^14^. Various adverse effects, including cough, throat or mouth irritation, headaches, and nausea, have also been reported^15^. In addition, respiratory symptoms such as asthma and chronic bronchitis have been observed in France among exclusive e-cigarette users, independently of conventional smoking and cannabis use^16^. Given the increasing use of e-cigarettes among adolescents and the concerns regarding their potential health risks, the present study aimed to assess the prevalence of e-cigarette use among high school students in Paris and to particularly explore adolescents’ motivations for e-cigarette use and their perceptions of its effects.

## Methods

### Study design and population

This was a descriptive, cross-sectional study, based on self-reported data via an online questionnaire, conducted among students enrolled in six educational institutions in the Paris region, including general, technological, and vocational high schools (*lycées*), as well as apprenticeship training centers (*centres de formation des apprentis*). Data collection took place between 22 January and 1 April 2021. Authorization to distribute the questionnaire to students was obtained from the administrations of the participating schools and, for private institutions, from parent association representatives. The study received ethical approval from the Research Ethics Committee of Université Paris Cité (N° 2020–92, on 15 December 2000), in accordance with the requirements of the Jardé Act. A data privacy declaration was submitted to the French Data Protection Authority (*Commission Nationale de l’Informatique et des Libertés*) on 6 January 2021. Informed consent was obtained from all participants at the beginning of the questionnaire.

### Questionnaire

The anonymized questionnaire was developed using the online survey platform LimeSurvey. The survey link was distributed to students by participating institutions via email or through their online learning platforms.

The self-administered questionnaire comprised 35 questions organized into five sections. 1) Demographic and medical characteristics, including age, self-declared sex, type of educational institution (general, technological, or vocational high schools [*lycées*], and apprenticeship training centers [*centres de formation des* apprentis]), and the existence of health problems, assessed by the question: ‘Do you have one or more health problems requiring regular medical treatment or follow-up?’ (yes, no), and categorized as ‘no reported health problem’ including asthma, psychiatric condition, or other health problem; 2) Tobacco-related variables. Experimentation was defined as at least one lifetime use. Current use included daily use (use at least once per day during the previous 30 days) and occasional use (non-daily use), with daily users considered a subgroup of current users. For tobacco use, variables included lifetime smoking (at least one cigarette), current smoking status (daily or occasional smoking during the previous 30 days), age at first cigarette, age at initiation of daily smoking, desire to quit smoking and attempts to quit during the previous 12 months, smoking among family members and friends, and heated tobacco use and knowledge; 3) E-cigarette use, including lifetime experimentation, current use (daily or occasional use at the time of the survey), and daily use (at least once per day during the previous 30 days). As the questionnaire did not capture detailed frequency among non-daily users, occasional users could not be further categorized according to vaping frequency. Additional e-cigarette-related variables included age at first use (in years), device ownership (yes, no), preferred device type (BOX, POD, TUBE, or no preference), use of nicotine-containing e-liquids, and e-cigarette use among family members and friends (yes, no); 4) Perceptions of e-cigarettes, assessed using binary agree/disagree responses, for items covering perceived harmfulness compared with conventional cigarettes, perceived nicotine content, addiction potential, financial burden, appealing smell and flavor, anxiety-reducing effects, and the discreetness and lightweight nature of e-cigarettes. This section was not administered to participants who had never used e-cigarettes; and 5) Motivations for e-cigarette use, assessed using binary agree/disagree responses, for items including curiosity, trendiness, intention to stop or reduce tobacco consumption, saving money compared with conventional cigarettes, practical considerations such as more discreet use, stress reduction, sensory appeal, passing time when bored, social influence, and perceived absence of addiction risk. This section was administered to all participants, including non-users.

### Statistical analysis

Continuous variables were summarized using means±standard deviations (SDs), medians, and ranges. Categorical variables were presented as frequencies and percentages. Group comparisons were performed using the chi-squared test or Fisher’s exact test when expected cell counts were <5. Prevalence ratios (PRs) and crude odds ratios (ORs) with their corresponding 95% confidence intervals (CIs) were calculated. In addition, to further explore associations while accounting for potential confounding, logistic regression analyses were performed. Adjusted ORs (AORs) and their 95% CIs were estimated after adjustment for age, sex, school type, and health status, which were considered potential confounding variables. Two-sided p<0.05 were considered statistically significant. All statistical analyses were performed using R software, version 4.0.5 (R Foundation for Statistical Computing, Vienna, Austria).

## Results

### Study population characteristics

A total of 1187 completed questionnaires were collected between 22 January and 1 April 2021, from students enrolled in six educational institutions in the Paris region, with a mean response rate of 17.9%, ranging from 10.4% to 25.9% across institutions. Participants were predominantly male (659/1187; 55.5%), with a median age of 17 years, and most were enrolled in general high school education (799/1187; 67.3%) (Table 1). Overall, 113 respondents (9.5%) reported at least one health condition, including asthma (43/1187; 3.6%) and psychiatric conditions, mainly depression (17/1187; 1.4%).

**Table 1:** Population characteristics and tobacco-related behaviors of adolescents included in a cross-sectional study conducted in six educational institutions in the Paris region, France, 2021 (N=1187)

Variable	Category	n (%)
**Sex**	Male	659 (55.5)
	Female	528 (44.5)
**Age** (years)	≤14	12 (1.0)
	15	208 (17.5)
	16	337 (28.4)
	17	399 (33.6)
	18	113 (9.5)
	19	59 (5.0)
	≥20	59 (5.0)
	Mean±SD	16.7±1.3
	Median (range)	17 (14–20)
**School type**	General high school	799 (67.3)
	Vocational high school	359 (30.2)
	Other	29 (2.4)
**Health status**	At least one reported health problem	113 (9.5)
	Asthma	43 (3.6)
	Psychiatric condition (mainly depression)	17 (1.4)
**Tobacco use**	Ever use of cigarettes	517 (43.6)
	Current tobacco use	277 (23.3)
	Daily tobacco use	149 (12.6)
**Heated tobacco product use**	Ever use of heated tobacco products	117 (9.9)
	Do not know about heated tobacco products	530 (44.7)
**Electronic cigarette use**	Ever use of electronic cigarettes	555 (46.8)
	Exclusive experimentation of electronic cigarettes	90 (7.6)
	Dual experimentation (tobacco and electronic cigarettes)	465 (39.2)
	Current use of electronic cigarettes	224 (18.9)
	Daily use of electronic cigarettes	102 (8.6)
	Do not know whether e-liquids contain nicotine	278 (23.4)

### Tobacco use

Overall, 517 students (43.6%) reported having ever used conventional cigarettes, with a mean age at initiation of 14.2±1.9 years. Current tobacco use was reported by 277 participants (23.3%), including 149 (12.6% of the total sample) who reported daily smoking. The mean age at transition to daily smoking was 15.6±1.7 years. Among the 277 current smokers, 214 (77.3%) had considered quitting, and 143 (51.6%) had attempted to quit during the previous 12 months. Heated tobacco products had been used at least once by 117 of the 1187 respondents (9.9%), while 530 respondents (44.7%) reported being unaware of these products. Among the 117 students who had used heated tobacco, 98 (83.8%) had also experimented with e-cigarettes.

### Electronic cigarette use

**Figure 1:**
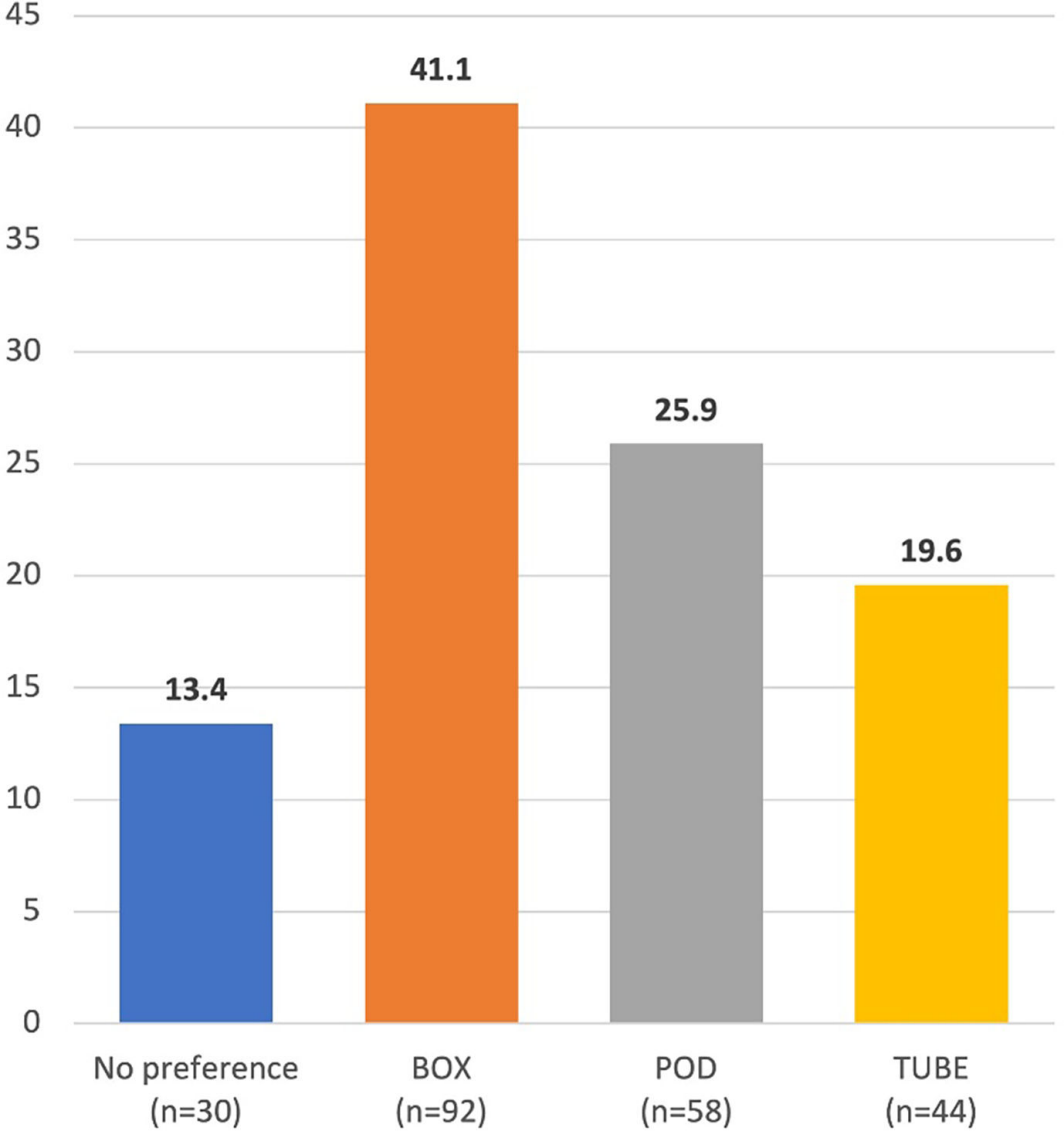
Preferred type (%) of electronic cigarette device among current users in a cross-sectional study of adolescents from six educational institutions in the Paris region, France, 2021 (N=1187; current users N=224)

A total of 555 students (46.8%) reported having ever used an e-cigarette, with a mean age at initiation of 14.9±1.9 years. Exclusive experimentation with e-cigarettes was reported by 90 respondents (7.6% of the total sample), while 465 (39.2%) reported dual experimentation with both conventional cigarettes and e-cigarettes. Among the 555 e-cigarette users, 351 (63.2%) did not own their own device and reported borrowing one. Current and daily e-cigarette use were reported by 224 (18.9%) and 102 (8.6%) of the 1187 respondents, respectively, while 62 respondents (5.2%) reported exclusive current e-cigarette use. Among the 224 current e-cigarette users, BOX models were the most commonly used device type (n=92; 41.1%) (Figure 1). E-cigarette ever users (n=555) and individuals who had never experimented with e-cigarettes (n=632) did not differ significantly by sex (p=0.16), but users were older (median age: 17 versus 16 years; p<0.05). Users were also less likely to be enrolled in general high school education (335/555 [60.4%] versus 464/632 [73.4%]; p<0.01) and more likely to attend vocational schools (209/555 [37.7%] versus 150/632 [23.7%]; p<0.01).

### Dual use of tobacco and electronic cigarettes

Overall, 465 respondents (39.2%) reported having experimented with both tobacco and e-cigarettes. Among these dual experimenters, 79 (17.0%) initiated e-cigarette use before tobacco, 180 (38.7%) initiated both in the same year, and 204 (43.9%) initiated e-cigarette use after tobacco. The mean interval between initiation of the two products was 0.6 years. Regarding current use patterns, 62 of the 1187 respondents (5.2%) reported exclusive e-cigarette use, 115 (9.7%) exclusive tobacco use, and 162 (13.6%) were dual users, concurrently using both products.

### Influence of social environment

Exposure to smoking or vaping within the social environment was significantly associated with both tobacco and e-cigarette experimentation, and these associations remained significant after adjustment for age, sex, school type, and health status. The strongest associations were observed for peer exposures. Adolescents with friends who smoked had nearly three times the prevalence of tobacco experimentation (PR=2.78; 95% CI: 2.32–3.32) and more than five-fold higher adjusted odds of experimentation (AOR=5.26; 95% CI: 4.02–6.94) compared to those without smoking friends. Similar associations were observed for e-cigarette experimentation (PR=2.71; 95% CI: 2.30–3.21; AOR=5.46; 95% CI: 4.20–7.13). Detailed PRs, ORs, and AORs for all social environment variables are presented in Table 2.

**Table 2:** Associations of exposure to smoking and vaping within the social environment with tobacco and e-cigarette experimentation among adolescents included in a cross-sectional study conducted in six educational institutions in the Paris region, France, 2021 (N=1187)

Exposure	Tobacco			E-cigarette		
	PR (95% CI)	OR (95% CI)	AOR (95% CI)	PR (95% CI)	OR (95% CI)	AOR (95% CI)
Family smoking	1.53 (1.35–1.74)	2.21 (1.74–2.80)	2.07 (1.61–2.66)	1.43 (1.27–1.61)	2.02 (1.60–2.57)	1.94 (1.52–2.48)
Friends smoking	2.78 (2.32–3.32)	5.42 (4.18–7.07)	5.26 (4.02–6.94)	2.71 (2.30–3.21)	5.75 (4.45–7.47)	5.46 (4.20–7.13)
Family vaping	1.34 (1.16–1.55)	1.76 (1.30–2.38)	1.58 (1.15–2.17)	1.29 (1.13–1.48)	1.69 (1.25–2.29)	1.59 (1.16–2.18)
Friends vaping	1.71 (1.49–1.96)	2.58 (2.04–3.27)	2.67 (2.08–3.43)	1.97 (1.72–2.25)	3.58 (2.82–4.56)	3.68 (2.88–4.73)

AOR: adjusted odds ratio. Adjusted for age, sex, school type, and health status.

PR: prevalence ratio.

### Current versus former e-cigarette users

Among the 555 adolescents who had ever experimented with e-cigarettes, 331 (59.6%) were former users and 224 (40.4%) were current users. In univariable analyses, exposure to smoking among friends was associated with current use (OR=2.61; 95% CI: 1.66–4.22; p<0.001), as was exposure to vaping among friends (OR=4.16; 95% CI: 2.78–6.33; p<0.001). In contrast, no significant associations were observed for smoking among family members (OR=1.14; 95% CI: 0.81–1.61; p=0.44) or vaping among family members (OR=0.87; 95% CI: 0.57–1.32; p=0.59). After adjustment for age, sex, school type, health status, and social environment variables, exposure to friends who used e-cigarettes remained independently associated with current use (AOR=3.98; 95% CI: 2.56–6.32; p<0.001).

### Perceptions of electronic cigarettes

**Figure 2:**
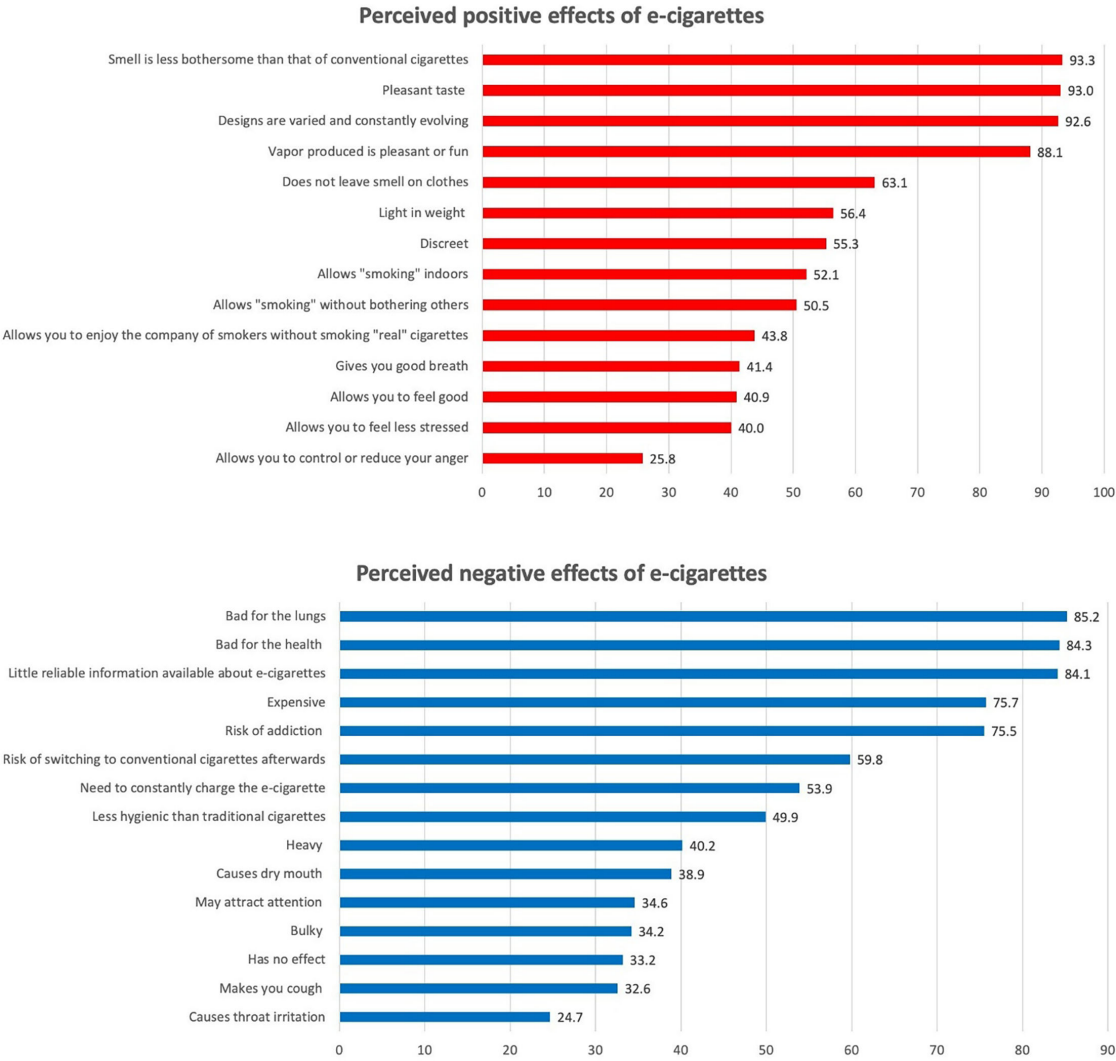
Agreement (%) with statements regarding perceived positive and negative effects of electronic cigarettes among ever users in a cross-sectional study of adolescents from six educational institutions in the Paris region, France, 2021 (N=1187; ever users N=555)

Of the 1187 respondents, 278 (23.4%) did not know whether e-liquids contained nicotine; this proportion remained notable among e-cigarette ever users (68/555; 12.3%). Most respondents perceived e-cigarettes as less harmful (541/1187; 45.6%) or equally harmful (449/1187; 37.8%) compared with conventional cigarettes, while 68 (5.7%) considered them more harmful. Figure 2 illustrates the perceived positive and negative effects reported by e-cigarette ever users (n=555). Positive perceptions of e-cigarettes were mainly related to sensory attributes, including a less bothersome smell than conventional cigarettes (518/555; 93.3%) and pleasant taste (516/555; 93.0%), as well as varied design (514/555; 92.6%) and enjoyable vapor experience (489/555; 88.1%). Negative perceptions included concerns about lung health (473/555; 85.2%), general health (468/555; 84.3%), lack of reliable information (467/555; 84.1%), expense (420/555; 75.7%), and addiction risk (419/555; 75.5%). A total of 332 respondents (59.8%) perceived a risk of transitioning to conventional cigarette use. Physical side effects such as dry mouth (216/555; 38.9%), cough (181/555; 32.6%), and throat irritation (137/555; 24.7%) were reported, along with practical inconvenience such as frequent charging (299/555; 53.9%).

### Motivations for electronic cigarette use

The levels of agreement with statements regarding motivations for e-cigarette use, stratified by e-cigarette use status (ever use, current use, and daily use), are presented in Table 3. Although these questions were administered to all participants, only responses from adolescents who had used e-cigarettes are reported.

**Table 3:** Agreement (%) with statements regarding motivations for using electronic cigarettes among electronic cigarette users, in a cross-sectional study of adolescents in six educational institutions in the Paris region, France, 2021 (N=1187)

**Motivation**	**Ever users** (N=555) n (%)	**Current users** (N=224) n (%)	**Daily users** (N=102) n (%)	**p**
Out of curiosity to try it	507 (91.4)	193 (86.2)	79 (77.5)	0.0010
For the variety of flavors available	416 (75.0)	186 (83.0)	82 (80.4)	0.0010
To be able to ‘smoke’ at home without being bothered by the smell of conventional cigarettes	353 (63.6)	180 (80.4)	89 (87.3)	0.0399
To stop or reduce my tobacco consumption	347 (62.5)	159 (71.0)	81 (79.4)	<0.001
To save money in the long-term (compared with conventional cigarettes)	313 (56.4)	150 (67.0)	78 (76.5)	<0.001
Out of consideration for others, to avoid exposing them to secondhand smoke and tobacco smell	287 (51.7)	128 (57.1)	63 (61.8)	0.6449
To pass time when bored	264 (47.6)	133 (59.4)	59 (57.8)	0.7033
Because of its playful or gadget-like aspect	224 (40.4)	90 (40.2)	33 (32.4)	<0.001
To manage stress	213 (38.4)	109 (48.7)	53 (52.0)	>0.90
To have better control over the ingredients in e-cigarettes compared with conventional cigarettes	162 (29.2)	83 (37.1)	53 (52.0)	0.0036
To be able to ‘smoke’ in places where smoking is prohibited because it is discreet	152 (27.4)	81 (36.2)	44 (43.1)	<0.001
Because friends or family members use e-cigarettes	138 (24.9)	58 (25.9)	28 (27.5)	0.2326
To overcome the difficulty of remaining a non-smoker	136 (24.5)	52 (23.2)	26 (25.5)	<0.001
Because the available information about e-cigarettes is reassuring	125 (22.5)	66 (29.5)	38 (37.3)	0.4058
Because there is no risk of addiction with e-cigarettes	81 (14.6)	45 (20.1)	22 (21.6)	>0.90
To be fashionable or trendy	75 (13.5)	25 (11.2)	9 (8.8)	<0.001
To express my personality because e-cigarettes can be customized	75 (13.5)	34 (15.2)	18 (17.6)	<0.001
To present a positive image of myself	25 (4.5)	10 (4.5)	5 (4.9)	0.0017

## Discussion

This cross-sectional study, conducted among 1187 high school students in the Paris region, revealed a high prevalence of e-cigarette experimentation, current use, and daily use. These findings are consistent with recent French national data reporting similar patterns of e-cigarette experimentation, current use, and daily use among high school students^1^. The mean age of e-cigarette initiation in our study was slightly higher than the mean age for tobacco initiation. This temporal pattern, combined with the finding that nearly one out of 5 dual experimenters tried e-cigarettes before conventional cigarettes, raises questions about the sequencing of product use. These observations are in line with data analyzed in a meta-analysis, which found that e-cigarette use is associated with increased odds of subsequent cigarette smoking initiation among adolescents and young adults^17^.

Our study also identified distinct motivational profiles that varied according to the frequency of e-cigarette use. Curiosity was the predominant motivation among ever users, consistent with previous research highlighting curiosity and the urge to try something new as key drivers of adolescent e-cigarette initiation^18^. However, among daily users, curiosity was less prominent, with more functional motivations predominating, particularly the ability to use e-cigarettes at home without tobacco odor and the availability of a variety of flavors. This shift from curiosity to more practical motivations, suggests that, as use becomes more regular, sensory and environmental factors increasingly contribute to maintaining e-cigarette use. Flavor variety (e.g. fruit and mint), which was also reported as a motivation by the majority of current users, has been consistently identified as an important driver of e-cigarette use among adolescents^19^. These findings underscore the importance of regulatory approaches targeting flavors in e-cigarette products, including bans or restrictions, to help prevent uptake among youth^20^. Furthermore, the observed prevalence of heated tobacco experimentation in the present study supports the need for similar surveillance and regulatory measures across all novel tobacco products^21^.

Exposure to tobacco or e-cigarette use within the social environment was strongly associated with experimentation, particularly when friends were users, reinforcing the influence of peer networks on adolescent behavior^22^. The finding that more than half of e-cigarette ever users did not own their own device and borrowed from others, also suggests that initial e-cigarette use often occurs in social contexts, further supporting the importance of peer influences^23^. Our results align with a meta-analysis of 21 studies showing that adolescents with family members or friends who smoke are more likely to use e-cigarettes^24^.

A concerning finding in our study was the notable lack of knowledge regarding e-liquid nicotine content, with nearly one quarter of respondents unaware whether the products they used contained nicotine. This knowledge gap persists, despite European regulations requiring mandatory nicotine content labeling, ingredient lists, and health warnings on e-cigarettes^25^. Importantly, a population-based study from the United States conducted among 821 adolescent and young adult e-cigarette users, found that this population has difficulty understanding nicotine concentrations labeled using the two most common metrics (mg/mL and percent nicotine), pointing out a need to simplify nicotine labeling and improve its clarity^26^.

Among current e-cigarette users, BOX devices were the preferred model in our study. This preference may contrast with findings from other studies reporting a predominance of POD-based systems, such as Puff Bar and JUUL, among adolescents, largely due to their discreet design and appealing flavors^5,7,27^ . This discrepancy may reflect the European regulatory context, where nicotine concentrations in e-liquids are capped at 20 mg/mL^4^. By contrast, JUUL products in the United States have been reported to contain higher nicotine concentrations, ranging from 22 to 56 mg/mL^−1^, typically in the form of nicotine salts (e.g. nicotine benzoate or levulinate) dissolved in propylene glycol and vegetable glycerin^6^. These regulatory differences may partly explain the lower use of declared POD-based devices in France.

### Strengths and limitations

This study has several strengths, including its relatively large sample drawn from six educational institutions with diverse academic pathways, enhancing the representativeness of different adolescent profiles. The comprehensive questionnaire allowed for a detailed assessment of motivations, perceptions, and use patterns, providing a nuanced understanding of e-cigarette use among adolescents in France. Although data collection took place during the COVID-19 pandemic, mobility restrictions were limited compared with the initial lockdown periods. From 21 March 2021, only minor measures were in place: secondary schools in the Île-de-France region operated at reduced capacity, and movement was permitted within a 10 km radius of one’s home without time restrictions. E-cigarettes, therefore, remained accessible to adolescents in school settings.

However, some limitations should be acknowledged. First, the cross-sectional design precludes any inference of causal relationships or temporal sequences between e-cigarette and tobacco use. Second, reliance on self-reported data may introduce misclassification, particularly regarding age at initiation and past behaviors. Third, the use of binary response options for perceptions and motivations did not allow assessment of the intensity of attitudes; Likert-scale measures would have provided greater granularity. Fourth, although multivariable analyses adjusted for several potential confounders, residual confounding cannot be excluded. Fifth, the modest and variable response rates across institutions raise the possibility of selection bias. Finally, as the study was conducted in the Paris region, the findings may not be generalizable to adolescents in other regions, particularly rural areas. Nevertheless, this study adds to the growing body of evidence on adolescent e-cigarette use by providing detailed insights into prevalence, motivations, and perceptions in a French context.

## Conclusions

E-cigarette use is prevalent among adolescents in the Paris region and is influenced by curiosity, sensory appeal, and social factors, particularly peer exposure. Our findings also highlight gaps in knowledge regarding nicotine content and emphasize the complexity of tobacco and e-cigarette use behaviors during adolescence. Additional studies, particularly longitudinal cohort studies, are needed to provide evidence-based information on tobacco and e-cigarette use trajectories among adolescents.

## References

[1] Spilka S, Philippon A, Le Nézet O, Janssen E (2026). Les usages de substances psychoactives chez les collégiens et les lycéens. Résultats EnCLASS 2024. Observatoire Français des Drogues et des Tendances Addictives (OFDT).

[2] Gebeyehu NA, Gelaw KA, Atalay YA (2025). Global prevalence of e-cigarette use among students: systematic review and meta-analysis. PLoS One.

[3] Baldassarri SR (2020). Electronic cigarettes: past, present, and future: what clinicians need to know. Clin Chest Med.

[4] Snell LM, Nicksic N, Panteli D (2021). Emerging electronic cigarette policies in European member states, Canada, and the United States. Health Policy.

[5] Fadus MC, Smith TT, Squeglia LM (2019). The rise of e-cigarettes, pod mod devices, and JUUL among youth: factors influencing use, health implications, and downstream effects. Drug Alcohol Depend.

[6] Goniewicz ML, Boykan R, Messina CR, Eliscu A, Tolentino J (2019). High exposure to nicotine among adolescents who use juul and other vape pod systems ('pods’). Tob Control.

[7] Wang TW, Gentzke AS, Neff LJ (2021). Characteristics of e-cigarette use behaviors among US youth, 2020. JAMA Netw Open.

[8] Cottin M, Catellin M, De Guiran E (2024). Understanding adolescent consumption patterns and attitudes towards the “puff” on the path to a smoke-free generation: a 2022 French perspective. Front Public Health.

[9] Cooper M, Harrell MB, Perry CL (2016). Comparing young adults to older adults in e-cigarette perceptions and motivations for use: implications for health communication. Health Educ Res.

[10] Patel D, Davis KC, Cox S (2016). Reasons for current e-cigarette use among U.S. adults. Prev Med.

[11] Raymond BH, Collette-Merrill K, Harrison RG, Jarvis S, Rasmussen RJ (2018). The nicotine content of a sample of e-cigarette liquid manufactured in the United States. J Addict Med.

[12] Gotts JE, Jordt SE, McConnell R, Tarran R (2019). What are the respiratory effects of e-cigarettes?. BMJ.

[13] Jin L, Lynch J, Richardson A (2021). Electronic cigarette solvents, pulmonary irritation, and endothelial dysfunction: role of acetaldehyde and formaldehyde. Am J Physiol Heart Circ Physiol.

[14] Rebuli ME, Rose JJ, Noël A (2023). The e-cigarette or vaping product use-associated lung injury epidemic: pathogenesis, management, and future directions: an official american thoracic society workshop report. Ann Am Thorac Soc.

[15] Lindson N, Butler AR, McRobbie H (2024). Electronic cigarettes for smoking cessation. Cochrane Database Syst Rev.

[16] Delmas MC, Pasquereau A, Renuy A (2024). Electronic cigarette use and respiratory symptoms in the French population-based constances cohort. Respir Med.

[17] Soneji S, Barrington-Trimis JL, Wills TA (2017). Association between initial use of e-cigarettes and subsequent cigarette smoking among adolescents and young adults: a systematic review and meta-analysis. JAMA Pediatr.

[18] Perikleous EP, Steiropoulos P, Paraskakis E, Constantinidis TC, Nena E (2018). E-cigarette use among adolescents: an overview of the literature and future perspectives. Front Public Health.

[19] King BA (2020). Flavors are a major driver of the youth e-cigarette epidemic. Am J Public Health.

[20] World Health Organization (May, 2025). Flavour accessories in tobacco products enhance attractiveness and appeal. https://cdn.who.int/media/docs/default-source/tobacco-hq/infosheet-flavouraccessories.pdf?sfvrsn=69cd4997_4&amp;download=true.

[21] World Health Organization (2020). Heated tobacco products: a brief. https://iris.who.int/server/api/core/bitstreams/a1b8f935-7e57-476b-84cb-1607a586ec11/content.

[22] Piombo SE, de la Haye K, Valente TW (2025). Network dynamics of social influence on e-cigarette use among an ethnically diverse adolescent cohort. Nicotine Tob Res.

[23] Alexander JP, Williams P, Lee YO (2019). Youth who use e-cigarettes regularly: a qualitative study of behavior, attitudes, and familial norms. Prev Med Rep.

[24] Wang JW, Cao SS, Hu RY (2018). Smoking by family members and friends and electronic-cigarette use in adolescence: a systematic review and meta-analysis. Tob Induc Dis.

[25] Yan D, Wang Z, Laestadius L (2023). A systematic review for the impacts of global approaches to regulating electronic nicotine products. J Glob Health.

[26] Morean ME, Wackowski OA, Eissenberg T, Delnevo CD, Krishnan-Sarin S (2021). Adolescents and young adults have difficulty understanding nicotine concentration labels on vaping products presented as mg/mL and percent nicotine. Nicotine Tob Res.

[27] Kong G, Bold KW, Morean ME (2019). Appeal of JUUL among adolescents. Drug Alcohol Depend.

